# Improving lifetime trajectories for vulnerable young children and families living with significant stress and social disadvantage: the early years education program randomised controlled trial

**DOI:** 10.1186/1471-2458-14-965

**Published:** 2014-09-17

**Authors:** Brigid Jordan, Yi-Ping Tseng, Nichola Coombs, Anne Kennedy, Jeff Borland

**Affiliations:** Department of Paediatrics, University of Melbourne, Melbourne, Australia; Clinical Sciences Theme, Murdoch Childrens Research Institute, Flemington Road, Parkville, 3052 Melbourne, Australia; Melbourne Institute of Applied Economic and Social Research, University of Melbourne, Melbourne, Australia; Faculty of Education, Charles Sturt University, Kragujevac, New South Wales 2795 Australia; Community Childcare Association Victoria, Preston South, 3072 Kragujevac, Victoria Australia; Department of Economics, University of Melbourne, Melbourne, Australia

**Keywords:** Randomised controlled trial, Toxic stress, Early years education, Child abuse and neglect, Vulnerable children

## Abstract

**Background:**

Children who experience neglect and abuse are likely to have impaired brain development and entrenched learning deficiencies. Early years interventions such as intensive education and care for these children are known to have the potential to increase their human capital. The Early Years Education Program (EYEP) is a new program offered by the Children’s Protection Society (CPS) in Melbourne, Australia. EYEP is targeted at the needs of children who have been or are at risk of being abused or neglected. It has the dual focus of seeking to address the consequences of abuse and neglect on children’s brain development and redressing their learning deficiencies. Our objective is to determine whether EYEP can improve school readiness by conducting a randomised controlled trial (RCT) of its impacts.

**Methods/Design:**

The RCT is being conducted with 90 participants (45 intervention and 45 control). Eligible children must be aged under three years and assessed as having two or more risk factors as defined in the Department of Human Services *Best Interest Case Practice Model*. The intervention group participate for three years (or until school entry) in EYEP. The trial does not provide any early years education or care to the control group. Data are being collected on outcome measures for participants in EYEP and the control group at the baseline, at yearly intervals for three years, and six months after commencing the first year of school. Outcome measures encompass children’s health and development, academic ability and emotional and behavioural regulation; and quality of parenting practices. The study will evaluate the impact of EYEP on these outcomes, and undertake a benefit-cost analysis of the program.

**Discussion:**

Findings from the study have the potential to influence the quality of care and education for the large population of children in Australia who are at risk of abuse and neglect, as well as for children in mainstream childcare. The study will provide up-to-date evidence on the impact of an early years intervention relevant to an urban population in Australia; as well as (to our knowledge) being the first RCT of an early years education and care intervention in Australia.

**Trial registration:**

ACTRN 12611000768998. Date 22^nd^ July 2011.

## Background

### The problem

Interpersonal experience is the primary influence on brain development in early life [1–3]. Caregivers therefore have a critical role in brain activation and must provide nurturing, protective, secure and consistent relationships to ensure a young child’s optimal development [4–6]. Exposure to physical, emotional and/or sexual abuse and traumatic experiences early in life may have profound adverse effects on brain development including emotion regulation capacities and ability to cope with stress [7, 8]. Entrenched neglect can impair all aspects of young children’s growth and development [9–12].

Disruption to brain development affects the ability to learn, with recent studies for example showing that self-regulation is linked to the development of literacy and numeracy skills [13]. Gaps between children in the development of cognitive and social skills early in life are likely to become entrenched in later years. This happens because skill development is dynamic and hierarchical. Children who miss out at an early age lack the necessary building blocks and foundation for subsequent learning [14, 15].

Deficiencies in cognitive and social skills that develop before the age of five are likely to become the basis of problems such as low education attainment, unemployment, teenage pregnancy, and involvement in crime. There is also some evidence that children with this background of neglect and abuse cause negative spill-over effects on learning to other children without that background with whom they associate [16]. The consequences of neglect and trauma extend to health. Early adversity has been linked to a variety of health-threatening behaviours in adolescence and adulthood [17]. It also has been shown to cause physiological disruptions that persist into adulthood and can lead to disease; an example is alterations in immune function [18, 19].

### Approaches to dealing with the problem

A variety of early years education and care programs targeted at increasing the human capital of disadvantaged children have been implemented. These programs have consisted primarily of intensive early years education and care and/or home visiting and parent education [20], pp41-51].

Notable examples are the Perry Preschool program and the Abecedarian Project [21], pp.115-16]. The Perry program provided one to two years of part-day education and weekly home visits for low-IQ African American children aged three to four years living in low-income households in Ypsilanti, Michigan, during the 1960s. The Abecedarian program provided year-round full-time center-based care for five years, starting from the first year of life, for mostly African American children from low-income households from Chapel Hill, North Carolina.

Programs that increase the human capital of children from disadvantaged backgrounds can benefit those individuals, and reduce inequality in childhood human capital development. Summary evidence on the impact of early years interventions is available from a meta-analysis of 84 studies of outcomes from programs implemented between the early 1960s and mid 2000s. Only studies that met minimum standards for quality of research methods were included in the review. The meta-analysis found an average effect size on cognitive and achievement scores of 0.35 standard deviations at the end of the treatment period. This effect decayed at a rate of 0.03 standard deviations per year after the end of treatment. Less evidence is at present available to evaluate the effect of early years programs on non-cognitive or behavioural outcomes; and findings are mixed [21]. Other reviews of studies of early years programs obtain similar findings, with somewhat stronger evidence for effects on behavioural outcomes [20, 22–26].

Early years interventions can also provide spillover benefits for society. By increasing the human capital of children from disadvantaged backgrounds, future expenditures that a society might otherwise be required to make are avoided [20], pp. xxiii-xxiv]. Endowing children with higher levels of skills is likely to improve their school outcomes, meaning that less money needs to be spent on remedial education services such as repeated grades or special education classes. Where better school performance brings a higher level of educational attainment and improved labour market outcomes, then the government may benefit from higher tax revenues and reduced outlays for social welfare programs. Furthermore, staying longer in education and a greater likelihood of being in employment will reduce contact with the criminal justice system and the costs that would otherwise be incurred by government. Studies of the benefit-cost to society of early years interventions have generally found positive outcomes. For example, a recent review of the Perry program found an internal rate of return of 6–10 percent [27]; and an earlier summary of findings from studies of several programs found benefit-cost ratios from 1.3 to 17.1 [20], pp109-11].

Some existing early years programs in Australia have provided the same type of interventions for children from disadvantaged backgrounds – having a focus on either educational and learning outcomes or a focus on parenting and socialisation outcomes [28]. Only limited work to evaluate these programs has been undertaken. What research is available suggests mixed outcomes [26]; although one quasi-experimental study of a centre-based intervention for children at risk has found a positive impact on the well-being of children and parents [29].

The Early Years Education Program (EYEP) is a new program offered by the Children’s Protection Society (CPS), an independent not-for-profit child welfare organisation based in the north-east of Melbourne, Australia. CPS designed the EYEP to be targeted at the particular needs of children who have been or are at risk of being abused or neglected, and who would commence in the program prior to 3 years of age. EYEP’s objective is to ensure that these children realise their full potential and arrive at school developmentally and educationally equal to their peers.

EYEP has a dual focus: addressing the consequences of abuse and neglect on children’s brain development, and redressing their learning deficiencies. Jack Shonkoff has argued that most current programs for children from disadvantaged backgrounds do not have this dual focus – instead they mainly provide enriched learning experiences for children and parenting education for mothers. He suggests that a better approach for redressing inequalities in skill development is to adopt the dual focus: ‘by linking high-quality pedagogy to interventions that prevent, reduce, or mitigate the disruptive effects of toxic stress on the developing brain’ [30], p.982]. It is exactly this task that EYEP is taking up.

EYEP employs a holistic model in early education and care. Key features are high staff/child ratios (1:3 for children under 3 years, and 1:6 for children over 3 years), qualified staff, a rigorously developed curriculum, and the use of relationship-based pedagogy. The program involves direct intervention with a child to address his or her identified needs, reverse developmental delays, and reduce the impact of risk factors and adverse events. An innovative feature of the program is a trans-disciplinary model which includes an education leader who has graduate qualifications in early childhood curriculum (supported by a part-time early childhood curriculum consultant), an in-house infant mental health consultant, and family support consultant. There is a focus on developing relational pedagogical strategies to reduce the behavioural and emotional dysregulation resulting from living in a situation of toxic stress to enable the children to be more available for learning.

The basis for **care** in EYEP is an attachment-focused, trauma informed, primary-care model which recognises the significance of respectful, responsive relationships for every child’s learning and development. Every child is allocated a key worker who is that child’s primary carer. Purposeful, warm greetings and a clear idea of the routines and opportunities of the day are essential components of the model, which help to give children a sense of security, predictability and consistency. The objective of the primary care model is to encourage the fostering of significant attachments for children who are likely to be experiencing disrupted and compromised attachment relationships in their home environments.

The **education** model in EYEP is a pedagogically-driven reflective teaching model that is child-focused and built on the *National Early Years Learning Framework* of ‘Belonging, Being and Becoming’ [31]. Each child has individual learning goals developed by the educators in partnership with families. Educators plan a curriculum using play-based approaches and intentional teaching to support each child’s learning and development across learning outcomes in the *Early Years Learning Framework*. Play-based approaches and intentional teaching support children to develop a positive sense of identity as they explore, create, imagine, solve problems, learn communication skills, and develop friendships. It is understood that how children learn is just as important as what they learn. Reflection on practice guides the on-going planning cycle. Educators document, monitor and assess each child’s learning over time.

The EYEP model actively engages with **parents** to encourage on-going participation in the program, as well as in enhancing their usage of all available health, educational and social services available, in order to improve outcomes for children. At-risk children and their families characteristically have concurrent barriers which affect their inclusion in early childhood services; for example, chaotic lifestyles; mental health and substance abuse issues; family violence; low levels of educational attainment; insecure housing arrangements; and antisocial behaviour. Consequently, EYEP aims to sustain parental involvement and minimize attrition levels by improving parental engagement in their children’s development. In order to achieve this, care team meetings incorporating parents and family support/child protection workers and the early years educators (primary worker for the child) will take place every 12 weeks. These meetings aim to identify, with parents, the goals and aims they would like to achieve for their children, which is then documented on agreement forms and reviewed every 12 weeks.

EYEP can be seen as addressing a variety of barriers that might otherwise exist to families taking advantage of supportive services – such as affordability; families beliefs that may place low priority on early education services; and inter-personal barriers including beliefs and attitudes on the part of service providers that might compromise engagement [32].

EYEP is designed for children who are at serious risk of, or who have experienced abuse and neglect and are already demonstrating problems in emotional and behavioural regulation, delays in development, and whose families struggle to participate in universal early education services. It can therefore be considered a tertiary level intervention, equivalent to critical intensive care in the health sector. For that reason we do not anticipate that all features of EYEP would be needed in every universal early education and care program. Rather, specific features of EYEP could potentially be implemented to different degrees, as needed, in universal and secondary early education and care services. Some aspects of EYEP, such as strategies for sustaining participation, and skilling educators to assist vulnerable children with emotional and behavioural regulation, will be relevant for all universal early education and care settings. This interpretation of the potential application of EYEP fits well with recent reviews which conclude that an ideal system of early education and care should be based on a strong and inclusive universal set of services, backed by a tiered system of services to be provided to those with extra needs [33].

### Aims and hypotheses

We aim to conduct a randomised controlled trial of EYEP, in order to determine whether this intervention can improve the school readiness of participants. We hypothesise that, compared to the control group, the benefits of participation in EYEP at the end of the intervention, and six months after commencing the first year of school (Prep grade), will include:

- Improved child outcomes
- Better health and development outcomes
- Demonstration of a higher level of academic ability and achievement
- Better emotional and behavioural regulation
- Parents of children
- Reduced incidence of poor parenting practices
- Increased engagement with neighbourhood and community services.

## Methods/Design

### Design

**Figure 1:**
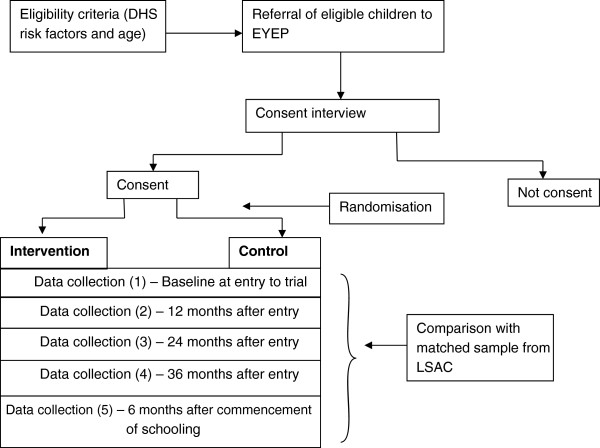
**Graphical depiction of components of the trial.**

The design of the randomised controlled trial of EYEP is shown in Figure 1. Eligible participants referred to the trial initially attend a consent interview. Those children whose parent or guardian consents to participate in the trial are randomly assigned between the intervention and control groups. Data collection occurs at the time of entry to EYEP, at 12, 24 and 36 months after entry, and at six months after commencement of schooling (Prep grade).

A pilot of the EYEP project was conducted in 2010 in order to hone the trial design, measurement methods, and the research process. Recruitment of trial participants commenced in 2011, and it is expected that recruitment will conclude in early 2015. Hence participation in EYEP by children involved in the trial will be complete by early 2018.

### Management, funding and ethics approval

The funding, implementation and management of EYEP has been undertaken by CPS. CPS formed an EYEP Research Committee (a sub-committee of the CPS Board) in late 2009 to manage the EYEP trial. The Research Committee is responsible for the design of the trial of EYEP and for monitoring implementation of the trial. The membership of the Committee is the researchers involved in the EYEP trial, Board members from CPS, the CEO of CPS and the Executive Director Services from CPS. The research group for the EYEP trial is multi-disciplinary. It includes researchers with expertise in infant mental health and social work, early childhood education, and program evaluation and statistical methods.

Places for children in EYEP and evaluation of the pilot have been funded by the Commonwealth Department of Department of Education, Employment and Workplace Relations (now Department of Education), and the Commonwealth Department of Families, Housing, Community Services and Indigenous Affairs (now Department of Social Services). Funding for places for children in EYEP and the research study has been provided by the Victorian Department of Human Services, Potter Foundation, Ross Trust, Pratt Foundation and Myer Foundation. Funding for the research study has been provided by VicHealth, the Crawford Foundation the Murphy-Nicols family, the Antipodean Family Foundation, and Australian Research Council Linkage Grant LP140100897.

The study protocol for the randomised controlled trial of EYEP was approved by the University of Melbourne Human Research Ethics Committee (HREC 1034236).

### Participants

To be eligible for participation in EYEP, children must be aged from 0 to 3 years, assessed as having two or more risk factors as defined in the Department of Human Services *Best Interest Case Practice Model*, currently engaged with family services or child protection services, and where early education is part of the child’s care plan. Risk factors include having teenage parents, parental substance abuse, parental mental health difficulties, and the presence of family violence. A full list of risk factors is available in the publication *Child Development and Trauma Guide*[34].

### Recruitment process

Referrals of potential participants to the EYEP trial are made by caseworkers from clients of child welfare services accessed through Child FIRST and Child Protection within the Victorian Department of Human Services. Referrals are from the north-east catchment area in Melbourne where CPS is located. The referrer advises the family of a child for whom an early years education service is appropriate for their case plan that there is a research trial of EYEP being conducted and offers to refer the family to the Senior Research Clinician (SRC) to receive further information about the trial. Referring agencies advise the family of a child being referred that there are a limited number of places available in EYEP and that allocation is being done by randomization. The SRC contacts the family to arrange a meeting with the Manager of EYEP and the SRC. At the meeting the trial is explained, and a Plain Language Statement is given to the parent(s). Written informed consent to participate in the trial, and to the process of random assignment, is then sought.

### Randomisation

After recruitment, a family is randomly assigned into either the EYEP participation group (intervention) or usual care group (control). Randomisation is done at arms-length. The random allocation into either the intervention or control group by order of recruitment into the trial is done by Dr Tseng, who then provides this information to Associate Professor Jordan in sealed opaque envelopes. When a family consents to participate in the trial the SRC calls the office of Associate Professor Jordan to have the envelope opened that will reveal the status of that participant. This is done in the presence of the family whose child is being randomised.

Blocked randomisation with a variable block size is being applied to conduct the random draw using the STATA sampling without replacement procedure. In families with multiple children participating in the trial all those children will be assigned to either the treatment or control group. When a new sibling of a participant is born during the recruitment phase, the SRC will determine current eligibility of the new sibling by liaising with family services or the child protection worker. When the eligibility conditions for participation in the trial are met, then the parent is invited to consent for the new infant to participate in the EYEP trial. New siblings recruited in this way are automatically allocated to the group (treatment or control) to which their older sibling has been assigned.

### Intervention and control group

The intervention group participate for three years (or until school entry) in EYEP. The control group receive what we refer to as ‘usual care’. This is a mix of parental and guardian care, and education and care provided by other local childcare centres or kindergartens. The usual care is determined by the choice of the child’s parent(s) without any direction; that is, there is no direct effect on the control group’s early education and care due to participation in the EYEP trial.

Nevertheless, it is possible that the control group may be affected by participation in the trial. For example, awareness of EYEP and its objectives may alter the decisions that parents of children in the control group make about their children’s early education. In addition, all children receive an annual development report which may change decisions that control group families make about their children’s needs and which services to access. Hence we plan to construct an extra quasi-experimental control group by matching trial participants with a group of children with similar background characteristics from the Longitudinal Survey of Australian Children (LSAC). By collecting some data items on outcomes for trial participants that are also collected for children in LSAC, we will be able to compare outcomes for the EYEP intervention and control groups with a control group from general population survey of children.

### Outcome measures

Data are being collected on participants in EYEP and the control group at the baseline (within 3 months of entry to the trial), at yearly intervals for three years after entry to the trial, and six months after commencing the first year of school (Prep grade).

Measures for data fields have been chosen on the basis of knowledge of international best-practice in measurement of child development. The measures have also been selected in order to enable comparisons with existing literature on child development and well-being and studies of previous trials such as Abecedarian, as well as to match with data items available for the alternative control group from the Longitudinal Survey of Australian Children. Table 1 lists the categories of data being collected on children and parents that relate to the main hypotheses in this study and the instruments being used.

**Table 1:** **Study measures and time points**

Study measure	Entry	Annual	School
**Hypothesis 1a) Children’s health and development outcomes**			
*Cognitive development*			
Bayley scales of infant and toddler development III (BSID)^a^	X	X	
Wechsler preschool and primary scale of intelligence (WPPSI) – Valid for ages 2.6-7.3^b^		X	X
*Language and socio-emotional development*			
Peabody Picture Vocabulary – Valid for ages 2 + ^c^		X	X
Child health questionnaire – Valid for ages 5+; Measures functional health and well-being^d^		X	X
Expressive Vocabulary Test		X	X
Longitudinal Study of Australian Children (LSAC) questions – Items on health status, injury and hospitalisation, health care access and affordability	X	X	X
**Hypothesis 1b) Children’s academic achievement and ability**			
Woodcock Johnson NU tests – Literacy and numeracy^e^			X
Devereux Early Childhood Assessment Program (DECA) – 37 observer rated positive behaviour items (subscales are initiative, self-control and attachment)^f^	X	X	X
**Hypothesis 1c) Children’s emotional and behavioural regulation**			
Greenspan socio-emotional growth chart (SEGC) – 35 item measure of socio-emotional milestones for ages 0–42 months^g^	X		
Brief infant toddler social emotional assessment (BITSEA) – 42 item parent completed screener for social-emotional/behavioural problems for ages 12–36 months^h^	X	X	
Child behaviour checklist – Identifies behavioural problems; Valid 18+ months^i^		X	X
Alarm baby distress scale (ADDB) – Social and interactive behaviour of infant in interaction with researcher; Valid for ages 1–36 months^j^	X		
**Hypothesis 2 Incidence of poor parenting**			
*Parent–child relationship*			
Strange situation procedure (SSP) – Conducted in an unfamiliar toy-filled room consisting of a structured sequence of brief episodes of separations and reunions from primary caregiver; Valid for 8–30 months^k^	X	X	
Story stem assessment – Uses dolls and narrative to measure the child’s representations of their attachment relationships; Valid for ages 3+ years^l^		X	X
Emotional availability scale (EAS) – 20 minutes free play between child and mother, coded for adult sensitivity, structuring, non-intrusiveness, non-hostility and child’s responsiveness; Valid for ages 0–7 years^m^	X	X	X
*Parenting, stress and attitudes*			
Home observation measurement of environment (HOME)–Semi-structured interview and direct observation of home environment by trained assessor to measure parent responsiveness and acceptance of the child, organisation of the environment, learning materials, parental involvement, variety of experience^n^	X	X	X
The parenting daily hassles scale – 20 items where parents rate the frequency and intensity/impact of experiences that can be a ‘hassle’ to parents^o^	X	X	X
Longitudinal Study of Australian Children (LSAC) Parenting Practices: Parent self-efficacy and harsh parenting scales	X	X	X
K6 – Measures non-specific psychological stress in adults^p^	X	X	X
Rand depression screener – 8 item self-report that screens for depressive and dysthymic disorders^q^	X	X	X
Longitudinal Study of Australian Children (LSAC) questions – Items on use of health and welfare services, employment and experience of neighbourhood	X	X	X

Notes for Table 1:

^a^Bayley N: *Bayley Scales of Infant and Toddler Development.* 3^rd^ edition. New York: The Psychological Corporation – Harcourt Brace Jovanovich; 2006.

^b^Wecshler D: *Wecshler Preschool and Primary Scale of Intelligence*. 3^rd^ edition. San Antonio TX: PsychCorp; 2004.

^c^Dunn L, Dunn D: *Peabody Picture Vocabulary Test*. San Antonio TX: PsychCorp; 2007.

^d^Waters E, Salmon L, Wake M, Wright M, Hesketh K: *Child Health Questionnaire Australian. Authorised Adaption of the Child Health Questionnaire*. Melbourne: Royal Children’s Hospital.

^e^Woodcock RW, McGrew KS, Mather N: *Woodcock-Johnston III*. Itasca IL: Riverside Publishing; 2001.

^f^LeBuffe PA, Naglieriri JA: **The Devereux Early Childhood Assessment (DECA): A measure of within-child protective factors in preschool children**. *NHSA Dialog: A Research-to-Practice Journal for the Early Childhood Field* 1999: **3**(1):75–80.

^g^Greenspan SI: *Greenspan Socio-Emotional Growth Chart: A Screening Questionnaire for Infants and Young Children*. San Antonio TX: PsychCorp; 2004.

^h^Carter AS, Briggs-Gowan M: *BITSEA: The Infant-Toddler and Brief Infant Toddler Socio-Emotional Assessment*. San Antonio TX: PsychCorp; 2005.

^i^Achenbach TM: *Integrative Guide to the 1991 CBCL/4-18, YSR and TRF Profiles*. Burlington VT: University of Vermont, Department of Psychology; 1991.

^j^Geudeney A, Fermanian J: **A validity and reliability study of assessment and screening for sustained withdrawal in infancy: The Alarm Distress Baby Scale**. *Infant Mental Health Journal* 2001: **22**(5):559–75.

^k^Ainsworth MDS, Blehar MC, Waters E, Wall S: *Patterns of Attachment*. Hillsdale NJ: Erlbaum; 1978.

^l^Emde RN, Wolf DP, Oppenheim D (eds): *Revealing the Inner Worlds of Young Children: The Macarthur Story Stem Battery and Parent–child Narratives*. New York NY: Oxford University Press; 2003.

^m^Biringen Z, Robinson J, Emde RN: *The Emotional Availability Scales*. 3^rd^ edition. Fort Collins CO: University of Colorado, Department of Human Development and Family Studies; 1998.

^n^Elardo R, Bradley R, Caldwell BM: **The relation of infants’ home environments to mental test performance from six to thirty-six months: A longitudinal analysis**. *Child Development* 1975: **46**:71–6.

^o^Crnic KA, Booth CL: **Mothers’ and fathers’ perceptions of daily hassles of parenting across early childhood**. *Journal of Marriage and the Family* 1991: **53**: 1043–50.

^p^Kessler RC, Barker PR, Colpe LJ, Epstein JF, Gfroerer JC, Hiripi E, Howes MJ, Normand ST, Manderscheid RW, Walters EE, Zaslavsky AM: **Screening for serious mental illness in the general population**. *Archives of General Psychiatry* 2003: **60**(2):184–9.

^q^Kemper KJ, Babonis TR: **Screening for maternal depression in pediatric clinics**. *American Journal Dis Child* 1992: **146**(7):876–8.

Data on background demographics and outcomes for children are being collected via standardized assessments, parent and childcare educator questionnaires, and observation and interviews. Data on parents and their outcomes – including background demographics, service usage (including childcare, counselling or social work services, usage of medical and hospital services), labour market experiences, and financial status (including income support payment history and financial stress) - is also being collected.

### Data analysis

The data analysis will identify the impact of ‘Intention to treat’ at the level of the individual child. The analysis will compare the change in an outcome measure (at time of data collection minus at baseline) for children in the intervention group with children in the control group. Impact evaluations will be conducted on outcomes measures after the 12, 24 and 36 month data collections as well following the data collection after the commencement of the first year of schooling.

For comparisons between the randomised intervention and control groups from the trial it should be possible to estimate the program impact as the difference in mean outcomes between those groups. For continuous outcome variables this will be the difference in mean changes in the outcome between intervention and control groups. For categorical outcome variables it will be differences in the change in the proportions of children in each category between intervention and control groups. For comparisons between the EYEP intervention group and the control group from LSAC a quasi-experimental matching method (difference-in-difference) will be used. Statistical significance will be established via t-tests for individual outcomes or Hotelling T tests for groups of outcomes.

### Economic evaluation

This analysis will quantify the benefits and costs of EYEP to society. Estimates of the net impacts of EYEP on children’s human capital development and service usage, and on outcomes for parents, will be translated into estimated monetary values, and these benefits aggregated to compare against the costs of the program, to arrive at a benefit-cost ratio for the program. The main potential benefits of the intervention are expected to derive from its effects on child health, cognitive development and interpersonal skills. Improved outcomes in these areas may yield benefits from: reduced direct cost to government such as reduced demands for health services, and reduced demand for specialized education due to better cognitive development. To assign monetary values to these benefits, we will for example collect information on the cost of health and special education services to estimate the values of reduced usage of those services. This information will then be combined with the estimated impacts of EYEP on service usage. The main costs associated with EYEP are the costs of providing the childcare and early education services; including employees’ wage costs, the cost of purchasing resources such as toys and educational materials, and maintenance costs for the program site. In undertaking the benefit-cost analysis of EYEP, it will be important to recognize what factors it may not be possible to value in monetary terms.

### Sample size and power calculations

The sample size has been determined taking into account both the numbers of observations needed to have sufficient power to detect likely effect sizes and the capacity of the CPS childcare centre where EYEP is being implemented. There is no previous Australian study of an intervention similar to EYEP, therefore calculation of the required sample size has been done using impact sizes estimated for the Abecedarian project. Impact sizes from the Abecedarian project are above those for most other early years programs [21], however, this can be attributed to its being an extensive and long-term intervention. The similar scale of intervention in EYEP makes Abecedarian an appropriate benchmark for this study. We use impact estimates from the Abecedarian project for IQ at 18 months and at 48 months ( [35] page 1918).

The sampsi routine in STATA was used to calculate the necessary sample size. When type one error is set at 0.05 and type two error set at 0.1 (power = 0.9), the minimum required sample size (per group) is 12 children based on the impact on IQ at 18 months IQ and 19 children based on the impact on IQ at 48 months. We then adjust this required sample size in two ways. First, we take into account that our sample will include families with multiple children. Based on the sample from the pilot phase of EYEP we estimate the number of children per family to be 1.35. We assume perfect correlation of outcomes between siblings which therefore requires expanding the minimum sample size by 1.35. Second, we incorporate an estimated attrition rate of 20 per cent per annum for the intervention and control groups (including item non-response). These adjustments result in a minimum sample size per group of 32 children. Hence it is expected that the sample size of 45 children in intervention and control groups will comfortably meet sample size requirements. It also provides a margin of safety in case standard errors in estimates of the impact of EYEP are larger than for the Abecedarian trial. All children in the Abecedarian trial were enrolled prior to reaching 6 months of age, while children recruited into the EYEP trial have been from a broader range of ages, up to 3 years. To the extent that age of enrolment is a source of variability in impact sizes, our estimates of impact size may therefore have larger standard errors than the Abecedarian study.

## Discussion

Abuse and neglect of children in their early years introduces long-term barriers to their development and impose substantial costs on a society. Hence the net gains to a society from remedying the consequences of that abuse and neglect are potentially large. EYEP is an innovative early years intervention, seeking as it does to address both the consequences of abuse and neglect on children’s brain development and to redress their learning deficiencies. This is the first RCT of an early years intervention of this intensity and duration that has this dual focus. Furthermore, there is a particular need for empirical evidence about the effects, and the relative costs and benefits, of early years education and care for at-risk children in Australia.

There is a large population of children in Australia for whom the EYEP is relevant. Using national data, it has been estimated that at any time there are 36,000 pre-school children in Australia in the same circumstances as the children eligible to participate in the EYEP trial, requiring casework services on the grounds of risk of child abuse and neglect [36, 37]. While the benefits of early and sustained education for vulnerable children may be well-established, currently those at-risk children who would benefit most seem least likely to have access to appropriate education and childcare in Australia. A recent report concluded that the Department of Education and Early Childhood Development ‘cannot demonstrate that early childhood services are accessible when and where needed, especially for vulnerable children’ [38]. Hence, the findings from the EYEP trial can influence policy for childcare and education that will affect a large national population.

The importance of having Australian studies of the impact of early childhood programs is that findings from international research cannot automatically be applied to Australia. This is because differences in the cultural context, family risk factors, and the family support service system between Australia and other countries, may affect how the program works. For example, participants in both the Perry and Abecedarian interventions were largely African-American, and lived in small cities (Ypsilanti and Chapel Hill). As well, the most well-known programs enrolled participants a long time ago, in the 1960s and 1970s, and were conducted in small cities. Doing the EYEP study will provide up-to-date evidence that is relevant to the urban environment in Australia.
